# What can hospital emergency admissions prior to cancer diagnosis tell us about socio-economic inequalities in cancer diagnosis? Evidence from population-based data in England

**DOI:** 10.1038/s41416-024-02688-6

**Published:** 2024-04-26

**Authors:** Aimilia Exarchakou, Bernard Rachet, Georgios Lyratzopoulos, Camille Maringe, Francisco Javier Rubio

**Affiliations:** 1 Inequalities in Cancer Outcomes Network (ICON), Department of Health Services Research and Policy, Faculty of Public Health and Policy, London School of Hygiene and Tropical Medicine, London, UK; 2 Epidemiology of Cancer Healthcare and Outcomes (ECHO), Department of Behavioural Science and Health, Institute of Epidemiology and Health Care, University College London, London, UK; 3 Department of Statistical Science, University College London, London, UK

**Keywords:** Epidemiology, Cancer epidemiology, Colon cancer

## Abstract

**Background:**

More deprived cancer patients are at higher risk of Emergency Presentation (EP) with most studies pointing to lower symptom awareness and increased comorbidities to explain those patterns. With the example of colon cancer, we examine patterns of hospital emergency admissions (HEAs) history in the most and least deprived patients as a potential precursor of EP.

**Methods:**

We analysed the rates of hospital admissions and their admission codes (retrieved from Hospital Episode Statistics) in the two years preceding cancer diagnosis by sex, deprivation and route to diagnosis (EP, non-EP). To select the conditions (grouped admission codes) that best predict emergency admission, we adapted the purposeful variable selection to mixed-effects logistic regression.

**Results:**

Colon cancer patients diagnosed through EP had the highest number of HEAs than all the other routes to diagnosis, especially in the last 7 months before diagnosis. Most deprived patients had an overall higher rate and higher probability of HEA but fewer conditions associated with it.

**Conclusions:**

Our findings point to higher use of emergency services for non-specific symptoms and conditions in the most deprived patients, preceding colon cancer diagnosis. Health system barriers may be a shared factor of socio-economic inequalities in EP and HEAs.

## Introduction

Emergency Presentation (EP) is one of the broad routes to cancer diagnosis in England and represents the diagnosis following an unplanned hospitalisation in the National Health Service (NHS).

Despite some improvement in recent years, colorectal cancer has one of the highest proportions of patients diagnosed through EP among all cancers diagnosed in England, at around 22%. Patients diagnosed through this route experience significantly lower survival than those diagnosed through the two-week wait or other referral routes [1, 2] and report worse patient experience [3–5]. While most relevant evidence relates to English patients, a recent international study indicated that diagnosis of colon cancer as an emergency is a global phenomenon [6].

EP is an indicator of delays in diagnosis and can be a manifestation of both patient-specific behavioural patterns such as recognition of symptoms or cancer awareness as well as problems in access to and delivery of health care services [7]. The importance of monitoring EP proportions in England, has been recognised and Routes to Diagnosis are now regularly reported as Official Statistics by the National Disease Registration Service at NHS Digital [1].

Wide inequalities in EP with colorectal cancer are observed, with older, more deprived, female patients, patients of non-white ethnicity background and patients with comorbidities at a higher risk for an emergency diagnosis [8–12]. The large proportion of EP, especially in the more socio-economically deprived groups, may reflect the overall increased relative use of emergency to elective hospital care in more deprived areas of England [13, 14]. The excess number of emergency hospitalisations in the more deprived patients can be only partly attributed to the severity of comorbidity prevalent in the more deprived areas, pointing to other systemic factors of care delivery [13].

In this study, we hypothesise that whether patients use elective or emergency route to be admitted in hospital in the years preceding their cancer diagnosis, is linked to EP. The conditions for which patients get hospitalised for and the admission route for those conditions, even if unrelated to the cancer, can help understand the use of healthcare services and problems of access in cancer patients. Benchmarking the disease-mix and the risk of emergency hospitalisation in most deprived patients against the least deprived cancer patients, can further highlight the inequalities component. For example, we know that patients with specific comorbidities are at a higher risk for EP [10] but it still remains unclear whether the type of admission for these conditions varies by socio-demographic characteristics.

Our study aims to understand the mechanism associated to HEAs, which in turn may help devise strategies to improve outcomes for the most affected population groups and reduce the overall burden of emergency presentation. With the example of colon cancer, we aim to tackle this by 1) examining whether more deprived patients and patients diagnosed through EP experience higher proportion of HEAs up to two years prior to colon cancer diagnosis and 2) identifying the combination of conditions or diseases that most commonly trigger the HEAs.

## Methods

### Data

We included all patients diagnosed with colon cancer (C18.0-C18.9) in England in 2013. Information on patient and tumour characteristics was retrieved from the English Cancer Registry Data. Whilst the Routes to Diagnosis is part of this dataset, the original information is derived through an algorithm developed by Public Health England using hospital records. With this algorithm, a cancer case is assigned to a route to diagnosis by examining the type of hospital admission on the date closest to the date of the cancer diagnosis, typically up to 28 days before the cancer diagnosis [2].

Patient history of hospitalisations was derived from the Admitted Patient Care (APC) records of the Hospital Episode Statistics (HES) in the two years preceding the cancer diagnosis [15, 16]. Patient admissions comprise of spells - periods of continuous care in one provider institution and each spell may comprise of more than one episode, i.e., a period of continuous care under the responsibility of a single consultant, although this is only a small proportion of hospital admissions (around 20%) [16].

The linkage of the cancer registry and HES datasets was deterministic, based on patient and tumour pseudo-identifiers and has been described in previous studies [12, 17]. We excluded 2,522 patients for whom there was no record in HES. We analysed the primary diagnostic codes, recorded in the first of 20 diagnostic fields, using the International Statistical Classification of Diseases, Injuries and Causes of Death, tenth revision (ICD-10) coding system [18]. The primary diagnostic codes are more likely to accurately represent the trigger cause for hospital admission and more likely to be linked to the mode of admission (emergency or non-emergency).

For the purpose of this study, we used the first diagnostic field of the discharge episode of the spell to define the reason for hospital admission. For the majority ( > 95%) of the multi-episode spells, the main diagnosis in the admission episode was identical to the main diagnosis in the discharge episode. The data selected for this study contained 5,118 distinct ICD-10 codes. As many individual diagnostic codes presented strong clinical or symptom similarities, we a priori grouped the codes into 58 aggregate condition groups. Furthermore, several grouped admission codes had low or zero incidence in certain combinations of sex and deprivation population groups and were excluded. The total number of aggregate condition groups initially considered for analysis was therefore 42.

Socioeconomic deprivation of patients was based on the income domain score of the English Index of Multiple Deprivation of the lower-layer super output area (LSOA) of patient residence at the time of cancer diagnosis [19]. Deprivation of cancer patients was then categorised according to the quintiles (from 1 indicating “least deprived” to 5 indicating “most deprived”) of the national distribution of scores for all LSOAs in England.

We only used the income domain as the IMD contains components about health deprivation and access to public services which are strongly related to inequalities in cancer outcomes, and therefore may lead to erroneous results and misinterpretation [20, 21]. Additionally, the income and employment domains have the highest degree of agreement with the overall composite IMD measure, as they carry the highest contributing weights (22.5%) [19].

### Data analysis

To describe trends in hospital admissions we present two main measures: the monthly rate of hospital admissions per patient and the monthly proportion of patients with at least one emergency admission. The latter represents unique patients, meaning that each patient can only be part of the monthly proportion once. These measures are useful to interpret trends in HEA over the 2-year pre-diagnostic interval and visualise differences between deprivation levels and routes to diagnosis (EP and non-EP). As most patients had a hospital admission on the date of colon cancer diagnosis, we excluded that hospitalisation from the visualization and analysis of hospital admissions (not from the descriptive table), to avoid the impact imbalanced data may have on the results. This led to the exclusion of 2,596 patients who had only one hospital admission during which the cancer diagnosis was made.

For the second study aim, i.e. identifying the combinations of conditions associated with HEAs, we developed a multi-step approach in order to select the relevant conditions among the very high number of admission codes recorded. The outcome was HEA (binary format) and the main predictors were the distinct grouped conditions and age at diagnosis. We fitted a generalized mixed-effects logistic model to account for the cluster structure of the observations at patient level. We specified a random intercept model with a logit link function implemented with the “glmer” function in R [22].

#### Multi-step approach to select the admission codes

For the selection of the most relevant groups of conditions, we adapted the Purposeful Variable Selection (PVS) for fixed effects logistic regression as described by Hosmer et al. (2013) [23], to mixed-effects logistic regression.

PVS represents a selection decision process in which, at each step, variables that are not significant and not a confounder are removed. At each step, the full model is compared to the nested model with a likelihood-ratio test (LRT) to determine the statistically significant covariates.

In Step 1, The PVS process began with a univariable analysis of each independent variable compared to the model with just the intercept. At the end of this step, all covariates that yielded a statistically significant *p* value were included to build the multivariable model M1.

In Step 2, M1 was compared to the nested model which included all but one of the M1 covariates. This was iteratively repeated for all covariates to determine the ones that could be eliminated, so that, at the end of Step 2, the reduced model M2 included only the covariates which were not eliminated.

In Step 3, any covariates eliminated in Step 2 were examined for confounding. Confounding was determined when a change in the remaining parameter estimates of model M2 compared to the parameter estimates of model M1 was greater than 10%. These confounders were added back to form the multivariable model M3.

In Step 4, each variable not selected in Step 1 was added one at a time to model M3 and its significance checked. At the end of this step, the final main effects model was obtained.

Due to large number of observations, we defined statistical significance based on *p* values less than 0.01 for LRT throughout the analysis. A few of the covariates (appendicitis, cognition and speech symptoms, musculoskeletal symptoms) created complete separation in specific combinations of sex and deprivation, because they perfectly predicted HEA or non-HEA. These were eventually removed from the mixed-effects model as they would otherwise create convergence issues in the Maximum Likelihood Estimation (MLE) and yield extremely large Wald standard errors (Hauck-Donner effect, i.e. the Wald test statistic is not monotonically increasing as a function of increasing distance between the parameter estimate and the null value) [24]. To detect complete separation we used the “brglm2” package in R only on the fixed effects [25]. All the analytic steps were stratified by sex and deprivation.

The marginal effect of each condition group retrieved from the final main effects model in Step 4 of the approach, using the R package “ggeffects” [26]. In the Supplementary material, we also present the average change in the probability of HEA that each covariate contributes at population level, alongside 95% approximate confidence intervals for the difference of two probabilities.

In the figures, a further clinical grouping of the conditions was done to facilitate the interpretation. The classification to “Potentially related”, “Indirect/non-specific” and “Unrelated” was done after the analysis, based on the similarity of colon cancer symptoms to the presenting symptoms of these conditions.

## Results

### Patient characteristics

The analysis included 15,263 patients diagnosed with a colon cancer in 2013 and who experienced at least one NHS hospital admission (HA) in the two-year window prior to colon cancer diagnosis (74% of all colon cancer cases). Approximately 15% of those patients lived in the most deprived and 21% in the least deprived neighbourhoods in England, at diagnosis. Although the majority of patients were aged over 65 years old at diagnosis in all deprivation groups, the distribution of age slightly shifted to younger ages with increasing deprivation (Table 1).

**Table 1:** Characteristics of male and female patients diagnosed with colon cancer in 2013, during the two years prior to diagnosis, by deprivation quintile^a^.

Male						
	Least deprived (*N* = 1722)	2 (*N* = 1725)	3 (*N* = 1687)	4 (*N* = 1611)	Most deprived (*N* = 1252)	Total (*N* = 7997)
Age in years (N (%))						
<45	51 (3.0%)	50 (2.9%)	45 (2.7%)	65 (4.0%)	56 (4.5%)	267 (3.3%)
(45–55]	69 (4.0%)	72 (4.2%)	86 (5.1%)	107 (6.6%)	92 (7.3%)	426 (5.3%)
(55–65]	242 (14.1%)	215 (12.5%)	248 (14.7%)	226 (14.0%)	232 (18.5%)	1163 (14.5%)
>65	1360 (79.0%)	1388 (80.5%)	1308 (77.5%)	1213 (75.3%)	872 (69.6%)	6141 (76.8%)
Patients with:						
1–2 HA^b^	1205 (70.0%)	1180 (68.4%)	1176 (69.7%)	1,094 (67.9%)	812 (64.9%)	5467 (68.4%)
3 HA	226 (13.1%)	228 (13.2%)	221 (13.1%)	225 (14.0%)	182 (14.5%)	1082 (13.5%)
>3 HA	291 (16.9%)	317 (18.4%)	290 (17.2%)	292 (18.1%)	258 (20.6%)	1448 (18.1%)
Patients with:						
1 HEA^c^	1433 (83.2%)	1432 (83.0%)	1375 (81.5%)	1302 (80.8%)	943 (75.3%)	6485 (81.1%)
2 HEA	173 (10.0%)	181 (10.5%)	168 (10.0%)	181 (11.2%)	166 (13.3%)	869 (10.9%)
>2 HEA	116 (6.7%)	112 (6.5%)	144 (8.5%)	128 (7.9%)	143 (11.4%)	643 (8.0%)

Female						
	Least deprived (*N* = 1500)	2 (*N* = 1642)	3 (*N* = 1516)	4 (*N* = 1467)	Most deprived (*N* = 1141)	Total (*N* = 7266)
Age in years						
(15–45]	56 (3.7%)	71 (4.3%)	74 (4.9%)	79 (5.4%)	77 (6.7%)	357 (4.9%)
(45–55]	71 (4.7%)	87 (5.3%)	87 (5.7%)	100 (6.8%)	74 (6.5%)	419 (5.8%)
(55–65]	186 (12.4%)	192 (11.7%)	190 (12.5%)	184 (12.5%)	186 (16.3%)	938 (12.9%)
>65	1187 (79.1%)	1292 (78.7%)	1165 (76.8%)	1104 (75.3%)	804 (70.5%)	5552 (76.4%)
Patients with:						
1–2 HA	1070 (71.3%)	1162 (70.8%)	1037 (68.4%)	1008 (68.7%)	733 (64.2%)	5010 (69.0%)
3 HA	196 (13.1%)	206 (12.5%)	195 (12.9%)	216 (14.7%)	169 (14.8%)	982 (13.5%)
>3 HA	234 (15.6%)	274 (16.7%)	284 (18.7%)	243 (16.6%)	239 (20.9%)	1274 (17.5%)
Patients with:						
1 HEA	1237 (82.5%)	1338 (81.5%)	1185 (78.2%)	1129 (77.0%)	851 (74.6%)	5740 (79.0%)
2 HEA	161 (10.7%)	173 (10.5%)	192 (12.7%)	206 (14.0%)	159 (13.9%)	891 (12.3%)
>2 HEA	102 (6.8%)	131 (8.0%)	139 (9.2%)	132 (9.0%)	131 (11.5%)	635 (8.7%)

^a^Based on the income domain score of the English Index of Multiple Deprivation.

^b^HA = Hospital Admissions.

^c^HEA = Hospital Emergency Admissions.

### Number of hospitalisations by route to diagnosis and deprivation

Excluding the hospitalisation during which the cancer diagnosis was made, patients had a total of 38,859 inpatient hospitalisations, 37% of which were emergency admissions. Approximately 80% of patients had at least one HEA and 20% had two or more HEAs.

**Fig. 1:**
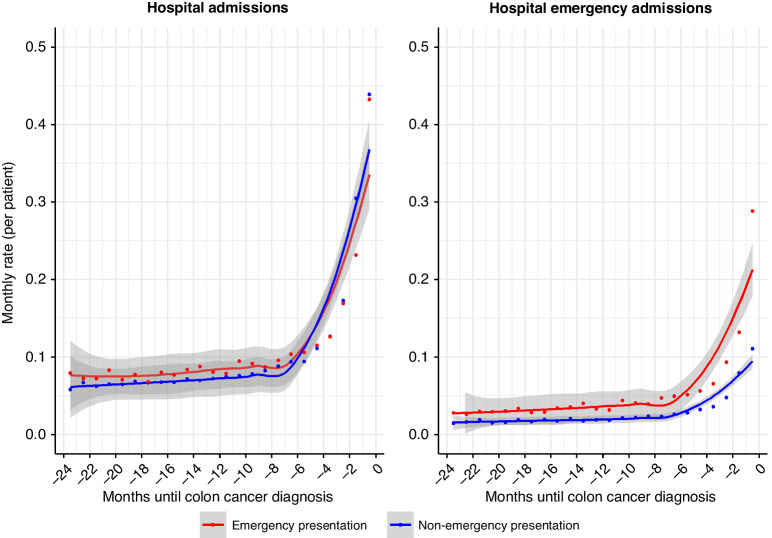
Monthly rate of hospitalisations in the two years prior to colon cancer diagnosis in patients diagnosed through emergency (*N* = 3671) and non-emergency presentation (*N* = 8996). Left panel: monthly rate of hospital admissions, right panel: monthly rate of hospital emergency admissions.

Patients whose colon cancer was diagnosed through emergency presentation (EP) had an excess of HEAs compared to those diagnosed through other routes (Non-EP) (Fig. 1). The difference in the rate of HEAs between the EP and Non-EP group of patients was relatively constant up to 7 months before diagnosis at around 0.01 difference. From 7 months onwards, the number of emergency admissions increased disproportionately for the EP group of patients, reaching a difference of 0.18 in the rate with the Non-EP group in the last month before diagnosis (0.29 emergency admissions per patient in EP group *vs*. 0.11 emergency admissions per patient in Non-EP group). The increase in hospital admissions (HA) occurred at the same time, around 7 months before colon cancer diagnosis, regardless the type (elective or emergency) of admission.

The proportion of patients with multiple HAs or multiple HEAs, increased with deprivation. Among male patients, 17% of the least deprived had more than three hospitalisations and 7% had more than two HEAs within two years prior to diagnosis (Table 1). In the most deprived, the proportions increased to 21% and 11%, respectively. Among female patients, 16% of the least deprived patients had more than three hospitalisations and 7% more than two HEAs, and these proportions rose to 21% and 14%, respectively, among the most deprived.

**Fig. 2:**
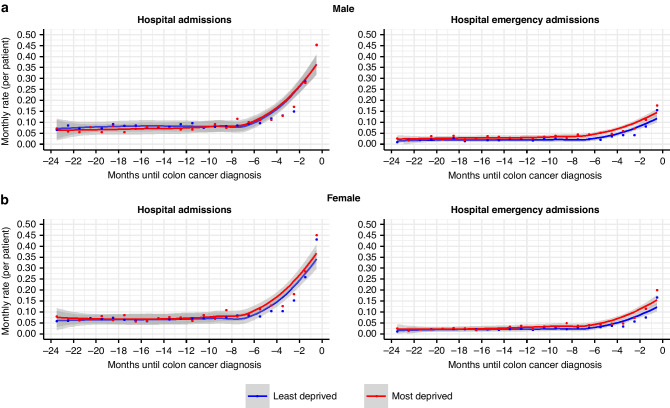
Monthly rate of hospitalisations in the two years prior to diagnosis in the most and the least deprived colon cancer patients. **a** Male. Left panel: monthly rate of hospital admissions; right panel: monthly rate of hospital emergency admissions. Most deprived *N* = 1040, least deprived *N* = 1407. **b** Female. Left panel: monthly rate of hospital admissions; right panel: monthly rate of hospital emergency admissions. Most deprived *N* = 956, least deprived *N* = 1234.

Deprivation-related differences were more marked for HEAs, where the rate was consistently higher in the most deprived than the least deprived patients (Fig. 2). These differences increased notably from 7 months before diagnosis, reaching a gap in monthly HEA rates of around 20% in the last month prior to diagnosis. Similar turning point is seen across the elective and non-elective hospitalisations, as in Fig. 1.

**Fig. 3:**
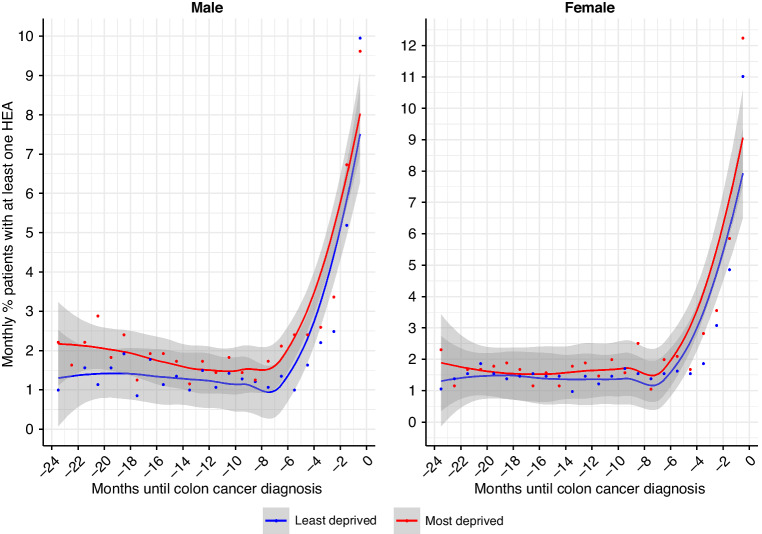
Proportion of colon cancer patients with at least one hospital emergency admission (HEA) in the two years prior to colon cancer diagnosis. Left panel: male; right panel: female.

The proportion of patients with at least one HEA, by contrast, was minimally higher in the most deprived than in the least deprived patients ( < 5% difference) (Fig. 3).

### Conditions predictive of emergency admission

**Fig. 4:**
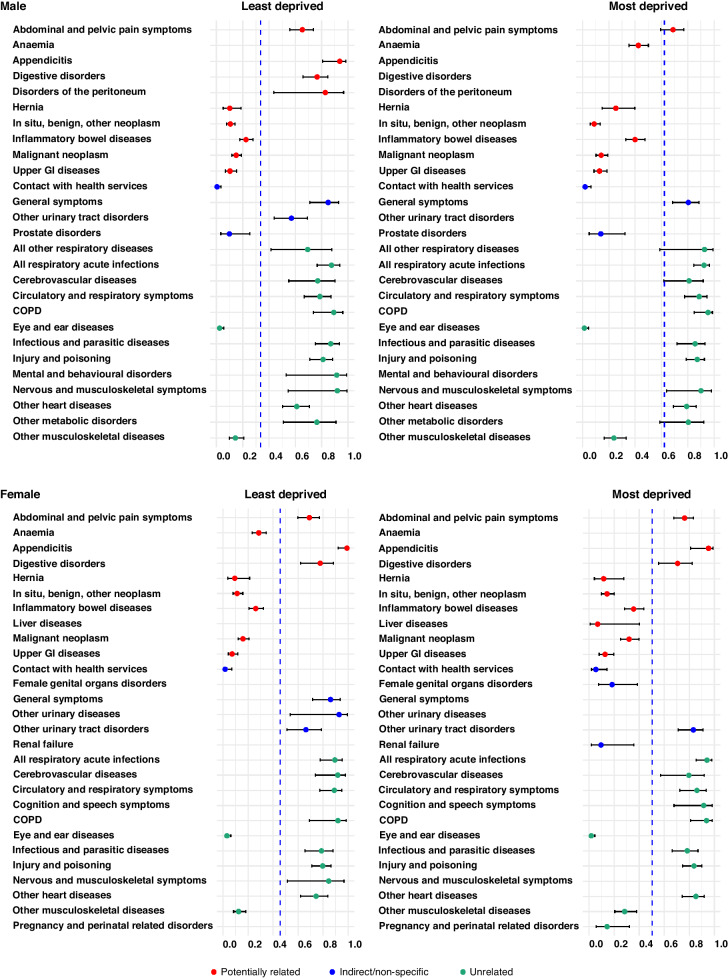
Marginal effects of the selected grouped conditions on the probability of Hospital Emergency Admission (HEA) in the two years prior to colon cancer diagnosis, by sex and deprivation level. The estimates are derived from mixed effect models specific to each of the four panels. Each of the four final models includes the set of covariates listed in each panel, as well as age. The vertical hyphenated blue line represents the average probability of HEA in the population, with all listed covariates set to zero and age at its mean value.

From the total of 42 clinical conditions initially included in each model, only 22–26 (varying by specific combinations of sex and deprivation) were retained in the final model as most predictive of the mode of hospital admission among colon cancer patients (Fig. 4 & Supplementary Table 2 & Supplementary Fig. 1).

Overall, some of the conditions with similar or potentially related symptoms with colon cancer such as abdominal and pelvic pain, appendicitis, digestive disorders and disorders of the peritoneum increased the probability of HEA above the baseline. In particular, appendicitis was highly predictive of HEA in both sexes and deprivation groups. In contrast, upper GI diseases and inflammatory bowel diseases decreased the probability of HEA below the baseline.

Urinary tract disorders and general symptoms also increased the probability of HEA. Patients with hospital admissions may present with non-specific symptoms that may be indirectly related to colon cancer.

From the group of conditions that are unlikely to be related to colon cancer, some acute conditions (e.g., infectious and parasitic diseases, injury and poisoning), some conditions affecting the cardiovascular and respiratory system (COPD, heart diseases) and mental and behavioural disorders increased the probability of HEA to higher than 0.6 in both male and female patients.

Cancer (malignant neoplasm, in situ/benign/other neoplasm) and some general conditions reduced the probability of HEA.

#### Discrepancies by sex and deprivation

The overall baseline probability of HEA for male patients, when age was set at its mean value (72.7 and 74.9 in the most and least deprived, respectively) and all grouped conditions were set to zero (reference values), was 0.62 (95%CI: 0.57–0.66) in the most deprived, almost double than in the least deprived (0.33; 95%CI: 0.3–0.37), despite their younger mean age (Fig. 4 & Suppl. Table 2 & Suppl. Fig. 1). In female patients (mean age at 72.8 and 75.2 in the most and least deprived, respectively), the baseline probabilities of HEA were similar in the two deprivation groups: 0.50 (95%CI: 0.45–0.55) in the most deprived and 0.45 (95%CI: 0.40–0.51) in the least deprived.

The marginal probabilities of HEA for individual conditions were similar across deprivation groups or sex. However, since the baseline probability varied widely between the most and the least deprived male patients, and between male and female patients, the average change in the marginal probabilities of HEA also appeared to vary.

Due to the higher baseline probability of HEA in the most deprived male patients, the specific conditions explained little of the HEA probabilities, as demonstrated by their smaller marginal effect than in the least deprived. In female patients, the marginal effect of individual conditions was similar between the least and most deprived patients, as their baseline probabilities were also very close.

Among male patients, the number of conditions that predicted type of admission was only 22 in the most deprived but 26 in the least deprived. There were very few discrepancies in which conditions predicted HEA between the two deprivation groups. Except for cardiovascular and respiratory conditions that were common, mental and behavioural disorders and some digestive conditions such as digestive disorders and disorders of the peritoneum, and urinary tract disorders all increased the probability of HEA above the baseline in the least but not in the most deprived.

In female patients, urinary diseases and general symptoms increased the probability of HEA in the least but not the most deprived patients. In contrast, gynaecological conditions such as pregnancy and perinatal related disorders, and female genital organs disorders increased the probability of HEA in the most but not the least deprived female patients.

Interestingly, anaemia was associated with a decrease in the probability of HEA only in the most deprived male patients, whilst among females the decrease was only in the least deprived patients.

## Discussion

While hospital admissions for any cause were very similar between the most and the least deprived colon cancer patients, emergency admissions preceding the diagnosis of colon cancer, clearly differed between socio-economic groups. This has been found internationally [27–29] as well as in the UK [30, 31].

The most deprived patients had an overall higher rate of HEAs and this was more marked in the last 7 months prior to cancer diagnosis. By contrast, the proportions of patients using the emergency services were fairly comparable between the most and the least deprived, at around 1.5% every month until 7 months before diagnosis.

Further, the patterns of grouped conditions as well as the level of their associated HEA probability were very comparable between most and least deprived patients, which suggests that there are similar conditions driving HEAs in both groups. Individual disease-specific aspects of care do not seem to explain the excess of emergency admissions in the most deprived patients, it may rather suggest an overall higher use of the emergency services as the privileged path. In other words, given the contrast between the proportion of patients (Fig. 3) and rate of HEA (Fig. 2) by deprivation, it seems that the higher HEA rate in the most deprived was mostly due to some patients using repeatedly the emergency services for conditions which were not well established.

Some digestive conditions such as appendicitis and to a lesser extent, abdominal and pelvic pain, represent a high proportion of HEA in colon cancer patients (Suppl. Table 1) and could be related to colorectal cancer. Appendicitis in older age groups could be a direct consequence of colorectal cancer potentially due to blockage of the appendix or stool obstruction. Abdominal and pelvic pain is another colorectal cancer symptom [32], often indicating late-stage tumour. Whilst appendicitis had similar effect on HEA probabilities in both the least and the most deprived patients, abdominal and pelvic pain represented higher proportion of HEA in the most than the least deprived.

Repeated use of emergency services by most deprived patients with abdominal/pelvic pain two years prior to definitive cancer diagnosis, suggests delays on the pathway to cancer diagnosis. Often, delays in cancer diagnosis are attributed to delays in seeking help due to lack of symptom awareness, limiting beliefs [33], underestimation of the seriousness of symptoms or increased comorbidities [34–37]. Whilst not minimising the impact of those factors, our study showed that there may be system-level factors that contribute to delays in diagnosis [38]. The extent to which the patient-related or the system-related factors account for EP with colorectal cancer is debatable and may vary by socio-demographic characteristics.

Against the cancer awareness hypothesis is the higher risk of EP for colon cancer in women [39]. Women generally have higher symptom awareness than men [40]. Nevertheless, our study showed that they had a higher baseline probability of HEA than men but similar marginal probabilities for the symptoms or conditions potentially related to colon cancer. Women also experience less specific symptoms which are more often attributed to benign diagnoses, which may explain some of their increased risk of EP for colon cancer. Abdominal symptoms such as changes in bowel habits are more likely to receive a benign diagnosis of IBS or diverticular disease than men, one year prior to emergency presentation with colon cancer [39].

Another example of sex and deprivation contrast, is anaemia, which was associated with HEA only in male patients and the least deprived female patients. This suggests that iron deficiency may be less promptly recognised or managed in more deprived female patients because any concurrent symptoms such as fatigue/lack of energy, pale skin, shortness of breath and headaches can be overlooked by the patient or the physician [41].

The conditions that increased the probability of HEA in the most and the least deprived colon cancer patients were a combination of acute and long-term conditions. Injury, poisoning, infectious and parasitic diseases and acute respiratory symptoms such as troubled breathing, persistent chesty cough and frequent chest infections, are all urgent care conditions and most likely require an emergency admission to the hospital. In contrast, long-term conditions or comorbidities such as malignant or benign neoplasm, COPD, heart diseases, renal disease and mental and behavioural disorders can be considered Ambulatory Care Sensitive Conditions (ACSC) for which HEA can be prevented [42–44].

In particular, among the more deprived colon cancer patients, a few of those ACSCs such as COPD, respiratory and heart diseases, were associated with a higher probability of HEA than in the less deprived. Also, conditions such as mental and behavioural disorders were only associated with HEA in the least deprived. Those discrepancies highlight the different disease burdens and severity of these conditions between deprivation groups and represent opportunities for preventing HEA, through the identification of vulnerable groups of patients [45].

Conditions such as malignant or benign neoplasm and upper GI related conditions were associated with decreased effect on the probability of HEA, i.e. they generally do not require HEA. For neoplasms, this may be because the majority of patients may return to the hospital for scheduled diagnostic or treatment appointments. Similarly, nearly 90% of the upper GI related conditions corresponded to inflammatory conditions or ulcer of the upper GI, with fairly specific symptoms and which are generally treatable in ambulatory setting. Most urinary disorders or infections increased the probability of HEA which aligns with the ACSCs statistics for England, reported as a quality indicator [46]. In recent years, a drop in the number of emergency admissions due to urinary tract infections was observed due to improved coding for sepsis [46].

EP flags challenges in early detection of cancer partly due to the disease itself e.g. rapidly progressing tumour, irregular or non-specific symptoms and complications that require emergency hospitalisation, but also due to patient help-seeking behaviours or other health-system factors related to the patient pathway [6]. Our findings add to the evidence that colon cancer EP and higher use of emergency services share similar drivers, particularly among the most deprived patients and those with more comorbidities [10, 13]. The groups of conditions in our study, do not seem to explain much of the HEAs among the most deprived, and they do not explain much of the inequalities in referral pathway either [47–49]. More deprived patients are less likely to be diagnosed with, and hospitalised for, symptoms and conditions related to colon cancer. In contrast, less deprived patients may opt to refer themselves to the Emergency Department for symptomatic but not critical conditions, bypassing the elective care system.

### Strengths and limitations

These analyses used population-based national cancer registrations known for their high level of completeness and quality [50]. These were successfully linked to secondary care records, as only 2,522 cancer patients (12%) did not have any HES record, most likely because they received care outside the NHS, such as privately or abroad.

The Purposeful Variable Selection method for confounding and covariate selection performs better than more automated methods when the analyst is interested in risk factor modelling rather than prediction, and this is especially true in smaller sample sizes [51]. However, one possible limitation is that the variables that were not selected initially for the multivariable model are only tested with the selected set of covariates one at a time and not jointly.

To assess the robustness of the method, we performed a sensitivity analysis using the “glmmmixedlasso” package in R, an ℓ1-penalized algorithm for fitting high-dimensional generalized linear mixed models [52]. The algorithm was applied to female patients of the least deprived group, testing a wide range of lambda values and evaluating them based on the acquired BIC values of each model. There was agreement with PVS in 24 out of the 29 variables retained except for “nervous system diseases”, “functional intestinal disorders”, “gallbladder and pancreatic diseases”, “abnormal and diagnostic imaging” and “skin disorders.” In contrast, “renal failure,” “other urinary diseases” and “liver diseases” retained with the PVS method were not retained in the sensitivity analysis. This result is potentially related to the false discovery problems associated to LASSO under highly correlated covariates.

To identify the combination of grouped conditions associated with higher probabilities of emergency admission, we further fitted a regression tree on the probabilities predicted from the final model. We used the “rpart” package in R [53]; The regression trees confirmed the absence of outstanding conditions or combinations of conditions that could be driving high probabilities of HEA. The overall patterns were again similar across deprivation and sex categories, only with fewer selected conditions among the most deprived male patients.

## Conclusion

To address inequalities in delays in cancer diagnosis, academic community and stakeholders have often focused on the lower cancer awareness and higher comorbidity prevalence observed in more deprived populations. Without ignoring these factors, our findings add evidence on an additional explanation. More disadvantaged populations may experience successive services-related barriers [54] in seeking help for any reason, causing delays in tests and diagnosis, and leading them to use emergency services [55]. For example, the current consultation conditions in primary care (e.g. short duration of consultation) [56] penalise the patients with poor health literacy, even more in the presence of multiple comorbidities [54]. Since the COVID-19 pandemic, patients with poor digital literacy may experience additional barriers due to the increased use of e-consultation. Delays may also occur in accessing diagnostic tests and specialised consultations [55]. Researchers and policymakers should shift their priorities toward the healthcare system factors that can influence these inequalities.

### Supplementary information

Supplemental Material

## Data Availability

The data used for this study are the English National Cancer Registry data and the Hospital Episode Statistics (HES). This data consists of patient information and as such, it is protected under the Data Protection Act 1998 and GDPR 2018 and cannot be made available as open data. Formal requests for release of the data can be made to the data custodian NHS Digital. The researchers will have beforehand obtained all the ethical and statutory approvals required for accessing sensitive data.
