# Supplementary material for: What can hospital emergency admissions prior to cancer diagnosis tell us about socio-economic inequalities in cancer diagnosis? Evidence from population-based data in England
Source: Br J Cancer. 2024 Apr 26;130(12):1960–8. doi: 10.1038/s41416-024-02688-6 (PMC11182764; doi:10.1038/s41416-024-02688-6)
Supplement: Supplementary file 1 — Supplemental Material [file 41416_2024_2688_MOESM1_ESM.docx]

**Supplementary material**

**What can hospital emergency admissions prior to cancer diagnosis tell us about socio-economic inequalities in cancer diagnosis? Evidence from population-based data in England.**

**Authors**

Aimilia Exarchakou^1^, Bernard Rachet^1^, Georgios Lyratzopoulos^2^, Camille Maringe^1^, Francisco (Javier) Rubio-Alvarez^3^

**Authors’ Affiliation**

^1^ Inequalities in Cancer Outcomes Network (ICON), Department of Non-Communicable Disease Epidemiology, Institute of Epidemiology and Health Care, London School of Hygiene and Tropical Medicine, London, UK

^2^ Epidemiology of Cancer Healthcare and Outcomes (ECHO), Department of Behavioural Science and Health, Faculty of Population Health Sciences, University College London, UK

^3^ Department of Statistical Science, University College London, UK

Table 1. Number and proportion of Hospital Admissions (HA) and Hospital Emergency Admissions (HEA) due to individual conditions, and proportions of HEA to HA due to individual conditions in the most and the least deprived colon cancer patients diagnosed in 2013.

| ICD-10 DISEASE GROUP | ICD-10 DIAGNOSTIC CODES | Least deprived | | | Most deprived | | |
| --- | --- | --- | --- | --- | --- | --- | --- |
|  |  | Number of **Hospital** Admissions  **(N=6,750)** | Number of **Emergency** Admissions  **(N=2,051)** | **%** | Number of **Hospital** Admissions  **(N=5,247)** | Number of **Emergency** Admissions  **(N=2,123)** | **%** |
| abdominal and pelvic pain symptoms | R1 | 246 (3.6%) | 143 (7%) | 58.1 | 224 (4.3%) | 145 (6.8%) | 64.7 |
| abnormal blood and urine findings | R7, R8 | 23 (0.3%) | 7 (0.3%) | 30.4 | 16 (0.3%) | 6 (0.3%) | 37.5 |
| abnormal diagnostic imaging | R9 | 53 (0.8%) | 6 (0.3%) | 11.3 | 47 (0.9%) | 13 (0.6%) | 27.7 |
| all other respiratory diseases | J (except other J) | 22 (0.3%) | 18 (0.9%) | 81.8 | 13 (0.2%) | 7 (0.3%) | 53.8 |
| all respiratory acute infections | J0-J3, J85-J86 | 102 (1.5%) | 89 (4.3%) | 87.3 | 127 (2.4%) | 114 (5.4%) | 89.8 |
| anaemia | D50-D64 | 645 (9.6%) | 213 (10.4%) | 33.0 | 519 (9.9%) | 190 (8.9%) | 36.6 |
| appendicitis | K35-K38 | 25 (0.4%) | 25 (1.2%) | 100 | 17 (0.3%) | 16 (0.8%) | 94.1 |
| cerebrovascular diseases | I60-I69 | 50 (0.7%) | 41 (2%) | 82.0 | 53 (1%) | 40 (1.9%) | 75.5 |
| chronic rheumatic heart diseases | I05-I09 | 6 (0.1%) | 2 (0.1%) | 33.3 | 1 (0%) | 0 (0%) | 0 |
| circulatory and respiratory symptoms | R0 | 131 (1.9%) | 105 (5.1%) | 80.2 | 109 (2.1%) | 94 (4.4%) | 86.2 |
| circulatory system diseases | I70-I99 | 129 (1.9%) | 55 (2.7%) | 42.6 | 119 (2.3%) | 58 (2.7%) | 48.7 |
| coagulatory defects | D65-D69, D7, D8 | 46 (0.7%) | 5 (0.2%) | 10.9 | 12 (0.2%) | 3 (0.1%) | 25.0 |
| cognition and speech symptoms | R4 | 19 (0.3%) | 19 (0.9%) | 100 | 26 (0.5%) | 24 (1.1%) | 92.3 |
| congenital malformations | Q | 8 (0.1%) | 2 (0.1%) | 25.0 | 7 (0.1%) | 1 (0%) | 14.3 |
| contact with health services | Z | 228 (3.4%) | 2 (0.1%) | 0.9 | 170 (3.2%) | 7 (0.3%) | 4.1 |
| COPD | J40-J47 | 54 (0.8%) | 47 (2.3%) | 87.0 | 86 (1.6%) | 80 (3.8%) | 93.0 |
| diabetes mellitus | E10-E14 | 11 (0.2%) | 7 (0.3%) | 63.6 | 19 (0.4%) | 12 (0.6%) | 63.2 |
| digestive disorders | K91-K93 | 125 (1.9%) | 94 (4.6%) | 75.2 | 91 (1.7%) | 58 (2.7%) | 63.7 |
| disorders of the peritoneum | K65-K67 | 7 (0.1%) | 5 (0.2%) | 71.4 | 7 (0.1%) | 5 (0.2%) | 71.4 |
| external causes of morbidity | V, W, X, Y, |  |  |  |  |  |  |
| eye and ear diseases | H | 388 (5.7%) | 16 (0.8%) | 4.1 | 311 (5.9%) | 8 (0.4%) | 2.6 |
| female breast disorders | N60-N64 | 1 (0%) | 0 (0%) | 0 |  |  |  |
| female genital organs disorders | N70-N74, N75-N98 | 41 (0.6%) | 9 (0.4%) | 22.0 | 28 (0.5%) | 4 (0.2%) | 14.3 |
| functional intestinal disorders | K57-K59 | 189 (2.8%) | 78 (3.8%) | 41.3 | 153 (2.9%) | 84 (4%) | 54.9 |
| gallbladder and pancreatic diseases | K80-K87 | 59 (0.9%) | 24 (1.2%) | 40.7 | 62 (1.2%) | 39 (1.8%) | 62.9 |
| general symptoms | R5, R6 | 80 (1.2%) | 65 (3.2%) | 81.3 | 118 (2.2%) | 81 (3.8%) | 68.6 |
| hernia | K40-K46 | 113 (1.7%) | 11 (0.5%) | 9.7 | 87 (1.7%) | 19 (0.9%) | 21.8 |
| hypertension | I10-I15 | 166 (2.5%) | 1 (0%) | 0.6 | 3 (0.1%) | 3 (0.1%) | 100 |
| hyperthyroidism | E05 |  |  |  |  |  |  |
| hypothyroidism | E02,E03 |  |  |  |  |  |  |
| in situ, benign, other neoplasm | D0-D4, D10-D36, D37-D48 | 446 (6.6%) | 32 (1.6%) | 7.2 | 304 (5.8%) | 20 (0.9%) | 6.6 |
| infectious and parasitic diseases | A, B | 97 (1.4%) | 76 (3.7%) | 78.4 | 95 (1.8%) | 73 (3.4%) | 76.8 |
| inflammatory bowel diseases | K50-K51 | 449 (6.7%) | 84 (4.1%) | 18.7 | 315 (6%) | 93 (4.4%) | 29.5 |
| injury and poisoning | S, T | 224 (3.3%) | 176 (8.6%) | 78.6 | 201 (3.8%) | 166 (7.8%) | 82.6 |
| ischaemic heart diseases | I20-I25 | 148 (2.2%) | 72 (3.5%) | 48.6 | 123 (2.3%) | 67 (3.2%) | 54.5 |
| liver diseases | K70-K77 | 36 (0.5%) | 11 (0.5%) | 30.6 | 24 (0.5%) | 8 (0.4%) | 33.3 |
| male genital organs disorders | N43-N51 | 11 (0.2%) | 3 (0.1%) | 27.3 | 7 (0.1%) | 3 (0.1%) | 42.9 |
| malignant neoplasm | C | 773 (11.5%) | 43 (2.1%) | 5.6 | 413 (7.9%) | 38 (1.8%) | 9.2 |
| mental and behavioural disorders | F | 14 (0.2%) | 12 (0.6%) | 85.7 | 24 (0.5%) | 17 (0.8%) | 70.8 |
| nervous and musculoskeletal symptoms | R25-R29 | 19 (0.3%) | 17 (0.8%) | 89.5 | 32 (0.6%) | 30 (1.4%) | 93.8 |
| nervous system diseases | G | 59 (0.9%) | 22 (1.1%) | 37.3 | 62 (1.2%) | 37 (1.7%) | 59.7 |
| obesity | E65-E68 |  |  |  | 1 | 0 | 0 |
| other heart diseases | I (except I05-I15, I20-I25, I60-I99) | 197 (2.9%) | 124 (6%) | 62.9 | 163 (3.1%) | 122 (5.7%) | 74.8 |
| other intestinal disorders | K52-K56, K60-K63 |  |  |  |  |  |  |
| other metabolic disorders | E0-E9 (except all other E) | 41 (0.6%) | 28 (1.4%) | 68.3 | 44 (0.8%) | 26 (1.2%) | 59.1 |
| other musculoskeletal diseases | M (except M30-M36) | 390 (5.8%) | 62 (3%) | 15.9 | 286 (5.5%) | 77 (3.6%) | 26.9 |
| other urinary diseases | N (except other N) | 11 (0.2%) | 6 (0.3%) | 54.5 | 21 (0.4%) | 14 (0.7%) | 66.7 |
| other urinary tract disorders | N20-N39 | 156 (2.3%) | 81 (3.9%) | 51.9 | 145 (2.8%) | 94 (4.4%) | 64.8 |
| pregnancy and perinatal related disorders | 0, P | 15 (0.2%) | 1 (0%) | 6.7 | 49 (0.9%) | 8 (0.4%) | 16.3 |
| prostate disorders | N40-N42 | 34 (0.5%) | 4 (0.2%) | 11.8 | 26 (0.5%) | 5 (0.2%) | 19.2 |
| renal failure | N17-N19 | 167 (2.5%) | 15 (0.7%) | 9.0 | 55 (1%) | 16 (0.8%) | 29.1 |
| skin disorders | L | 77 (1.1%) | 26 (1.3%) | 33.8 | 57 (1.1%) | 30 (1.4%) | 52.6 |
| skin symptoms | R20-R23 | 8 (0.1%) | 7 (0.3%) | 87.5 | 6 (0.1%) | 3 (0.1%) | 50.0 |
| special codes | U |  |  |  |  |  |  |
| systemic connective tissue disorders | M30-M36 | 1 (0%) | 0 (0%) | 0 | 1 (0%) | 1 (0%) | 100 |
| thyroid disorders | E04, E07 |  |  |  |  |  |  |
| thyroiditis | E06 |  |  |  |  |  |  |
| upper GI diseases | K0-K3 | 340 (5%) | 36 (1.8%) | 10.6 | 323 (6.2%) | 45 (2.1%) | 13.9 |
| urinary system symptoms | R3 | 70 (1%) | 24 (1.2%) | 34.3 | 45 (0.9%) | 19 (0.9%) | 42.2 |

Table 2. Marginal effects of the selected grouped conditions on the probability of HEA in the two years prior to colon cancer diagnosis

|  | Male | | Female | |
| --- | --- | --- | --- | --- |
| ICD-10 Disease Group | **Least deprived**  Baseline prob:  0.33 (95%CI: 0.3-0.37) | **Most deprived**  Baseline prob: 0.62 (95%CI: 0.57-0.66) | **Least deprived**  Baseline prob:  0.45 (95%CI: 0.4-0.51) | **Most deprived**  Baseline prob: 0.50 (95%CI: 0.45-0.55) |
| abdominal and pelvic pain symptoms | 0.653 (0.557-0.738) | 0.688 (0.593-0.770) | 0.689 (0.596-0.77) | 0.763 (0.676-0.833) |
| all other respiratory diseases | 0.693 (0.415-0.878) | 0.929 (0.587-0.992) | 0.894 (0.777-0.953) | 0.942 (0.856-0.978) |
| all respiratory acute infections | 0.876 (0.766-0.938) | 0.925 (0.847-0.965) |  |  |
| anaemia |  | 0.425 (0.353-0.500) | 0.285 (0.232-0.345) |  |
| appendicitis | 0.939 (0.809-0.982) |  | 0.989 (0.92-0.999) | 0.954 (0.811-0.99) |
| cerebrovascular diseases | 0.77 (0.549-0.902) | 0.810 (0.617-0.919) | 0.917 (0.738-0.978) | 0.797 (0.571-0.921) |
| circulatory and respiratory symptoms | 0.787 (0.668-0.871) | 0.888 (0.776-0.947) | 0.890 (0.774-0.95) | 0.862 (0.726-0.936) |
| contact with health services | 0.005 (0.001-0.034) | 0.020 (0.006-0.063) | 0.017 (0.004-0.07) | 0.054 (0.019-0.144) |
| COPD | 0.893 (0.736-0.961) | 0.955 (0.851-0.987) | 0.918 (0.691-0.983) | 0.938 (0.811-0.982) |
| digestive disorders | 0.766 (0.659-0.847) |  | 0.776 (0.619-0.881) | 0.706 (0.554-0.823) |
| disorders of the peritoneum | 0.828 (0.435-0.968) |  |  |  |
| eye and ear diseases | 0.025 (0.011-0.053) | 0.014 (0.004-0.045) | 0.031 (0.016-0.061) | 0.018 (0.007-0.045) |
| female genital organs disorders |  |  |  | 0.183 (0.074-0.385) |
| general symptoms | 0.850 (0.711-0.929) | 0.805 (0.684-0.887) | 0.859 (0.715-0.937) |  |
| hernia | 0.100 (0.052-0.185) | 0.253 (0.148-0.398) | 0.092 (0.037-0.212) | 0.116 (0.042-0.278) |
| in situ, benign, other neoplasm | 0.104 (0.076-0.141) | 0.087 (0.057-0.133) | 0.111 (0.077-0.157) | 0.142 (0.098-0.201) |
| infectious and parasitic diseases | 0.869 (0.753-0.935) | 0.857 (0.719-0.933) | 0.786 (0.654-0.877) | 0.784 (0.664-0.87) |
| inflammatory bowel diseases | 0.224 (0.178-0.278) | 0.400 (0.329-0.475) | 0.261 (0.207-0.324) | 0.356 (0.283-0.436) |
| injury and poisoning | 0.811 (0.71-0.883) | 0.873 (0.788-0.927) | 0.796 (0.708-0.863) | 0.838 (0.748-0.9) |
| liver diseases |  |  |  | 0.067 (0.008-0.401) |
| malignant neoplasm | 0.149 (0.116-0.19) | 0.141 (0.102-0.192) | 0.158 (0.119-0.207) | 0.32 (0.251-0.398) |
| mental and behavioural disorders | 0.916 (0.530-0.991) |  |  |  |
| nervous and musculoskeletal symptoms | 0.92 (0.546-0.991) | 0.902 (0.639-0.979) | 0.844 (0.514-0.965) |  |
| other heart diseases | 0.610 (0.502-0.708) | 0.792 (0.692-0.866) | 0.743 (0.618-0.838) | 0.853 (0.743-0.921) |
| other metabolic disorders | 0.764 (0.509-0.91) | 0.805 (0.586-0.923) |  |  |
| other musculoskeletal diseases | 0.143 (0.097-0.206) | 0.239 (0.165-0.332) | 0.124 (0.083-0.18) | 0.284 (0.205-0.378) |
| other urinary diseases |  |  | 0.928 (0.534-0.993) |  |
| other urinary tract disorders | 0.569 (0.438-0.691) |  | 0.661 (0.511-0.785) | 0.833 (0.71-0.911) |
| pregnancy and perinatal related disorders |  |  |  | 0.143 (0.055-0.322) |
| prostate disorders | 0.097 (0.033-0.254) | 0.138 (0.051-0.323) |  |  |
| renal failure |  |  |  | 0.094 (0.019-0.358) |
| upper GI diseases | 0.102 (0.066-0.154) | 0.128 (0.086-0.186) | 0.071 (0.042-0.118) | 0.127 (0.08-0.196) |

***The estimated probabilities are derived from mixed effect models specific to each of the four combinations of sex and deprivation. Each of the four final models includes the set of covariates listed in the relevant column, as well as age.***

***Fig. 1*** Average change in the probability of Hospital Emergency Admission in the two years prior to colon cancer diagnosis in the presence of each of the selected risk factors, by sex and deprivation


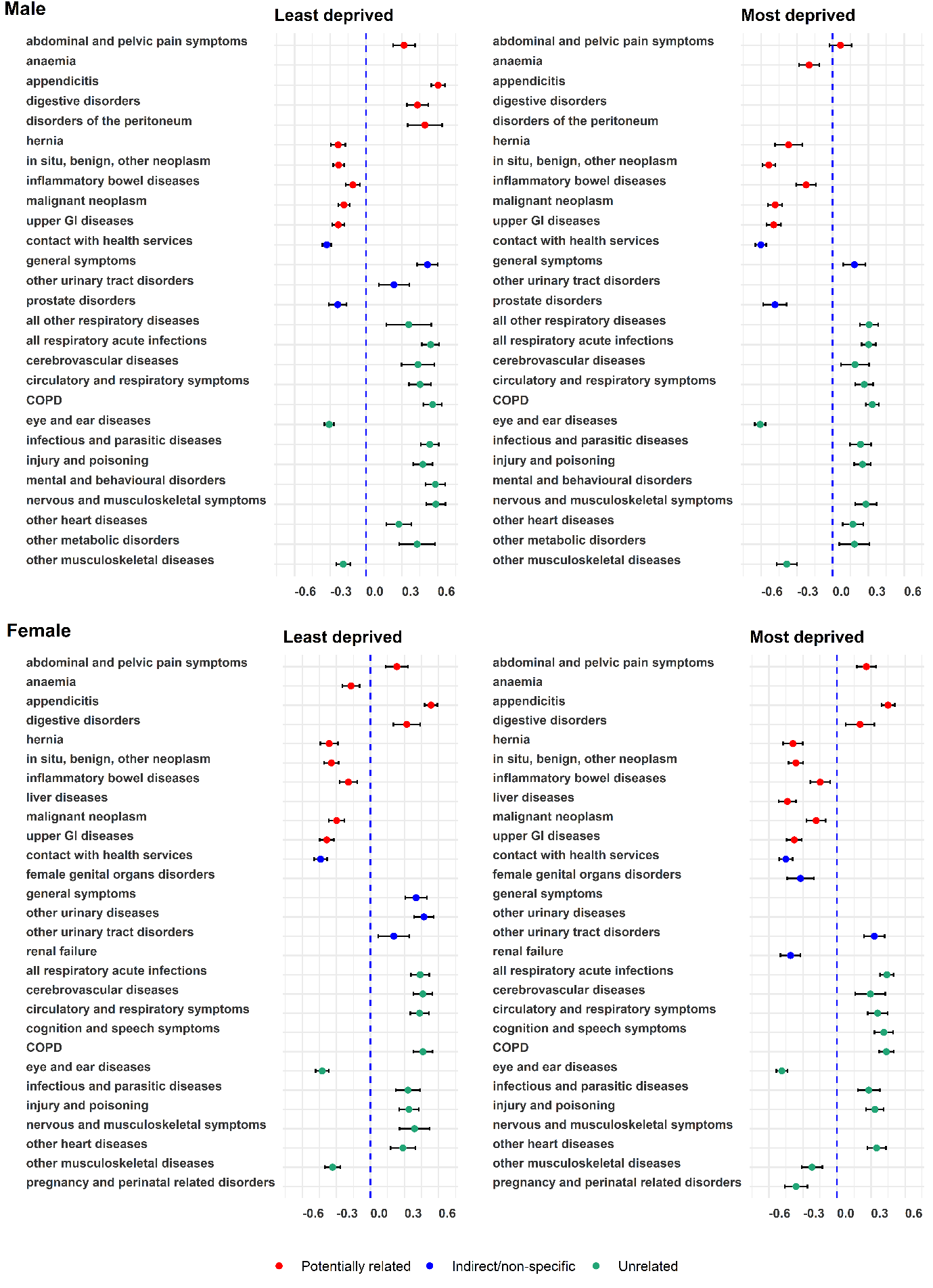


***The values on the figure represent the linear transformation of the probabilities retrieved from the mixed effect models specific to each of the four panels. Each of the four final models includes the set of covariates listed in each panel as well as age. The vertical hyphenated blue line represents zero effect.***
